# Inhibitory Control in the Absence of Awareness: Interactions Between Frontal and Motor Cortex Oscillations Mediate Implicitly Learned Responses

**DOI:** 10.3389/fnhum.2021.786035

**Published:** 2021-12-22

**Authors:** Silvia L. Isabella, J. Allan Cheyne, Douglas Cheyne

**Affiliations:** ^1^Program in Neurosciences and Mental Health, Hospital for Sick Children Research Institute, Toronto, ON, Canada; ^2^Department of Psychology, University of Waterloo, Waterloo, ON, Canada; ^3^Institute of Medical Sciences and Institute of Biomedical Engineering, University of Toronto, Toronto, ON, Canada; ^4^Department of Medical Imaging, University of Toronto, Toronto, ON, Canada

**Keywords:** frontal theta, sensorimotor gamma, cognitive control, oscillations, phase coherence, implicit learning, magnetoencephalography, pupillometry

## Abstract

Cognitive control of action is associated with conscious effort and is hypothesised to be reflected by increased frontal theta activity. However, the functional role of these increases in theta power, and how they contribute to cognitive control remains unknown. We conducted an MEG study to test the hypothesis that frontal theta oscillations interact with sensorimotor signals in order to produce controlled behaviour, and that the strength of these interactions will vary with the amount of control required. We measured neuromagnetic activity in 16 healthy adults performing a response inhibition (Go/Switch) task, known from previous work to modulate cognitive control requirements using hidden patterns of Go and Switch cues. Learning was confirmed by reduced reaction times (RT) to patterned compared to random Switch cues. Concurrent measures of pupil diameter revealed changes in subjective cognitive effort with stimulus probability, even in the absence of measurable behavioural differences, revealing instances of covert variations in cognitive effort. Significant theta oscillations were found in five frontal brain regions, with theta power in the right middle frontal and right premotor cortices parametrically increasing with cognitive effort. Similar increases in oscillatory power were also observed in motor cortical gamma, suggesting an interaction. Right middle frontal and right precentral theta activity predicted changes in pupil diameter across all experimental conditions, demonstrating a close relationship between frontal theta increases and cognitive control. Although no theta-gamma cross-frequency coupling was found, long-range theta phase coherence among the five significant sources between bilateral middle frontal, right inferior frontal, and bilateral premotor areas was found, thus providing a mechanism for the relay of cognitive control between frontal and motor areas via theta signalling. Furthermore, this provides the first evidence for the sensitivity of frontal theta oscillations to implicit motor learning and its effects on cognitive load. More generally these results present a possible a mechanism for this frontal theta network to coordinate response preparation, inhibition and execution.

## Introduction

Human behaviour is argued to be to be under both cognitive and automatic control, as articulated in dual-process theories (e.g., Evans and Stanovich, 2013). Within this framework, cognitive processes have been defined as mental acts of which we are conscious, that we intend, that require effort, and that can be controlled (Logan and Cowan, 1984). In contrast, automatic processes are rapid and autonomous, and are thought to yield default responses unless intervened on by cognitive processes. Both processes occur during inhibitory control, often employed in experiments using rapid response tasks that produce automatic responding requiring occasional intervention by cognitive processes, such as in a Go/No-Go or a Go/Switch task (e.g., Isabella et al., 2015). Go trials quickly become automatic, whereas No-Go or Switch responses require infrequent, intermittent inhibition of the prepotent Go response in favour of the alternate (No-Go or Switch). Such cognitive processes are often associated with activity in the frontal cortex. More precise neural mechanisms underlying these processes, however, remain unknown.

There are two signals in the frontal cortex that are often associated with inhibitory processes underlying cognitive control: evoked responses and oscillations. Frontal midline evoked responses represent brain activity that is phase-locked to a stimulus or response via “bottom-up” processes. In contrast, low-frequency frontal theta oscillations (4–8 Hz) represent ongoing brain activity that is not phase-locked to the stimulus or response via “top-down” processes. These two signals may be related in that evoked responses are discrete events that can be caused by shifts in ongoing oscillatory activity (such as baseline, amplitude or phase shifts) across several frequency bands, including and especially alpha (8–12 Hz) (Nikulin et al., 2007; Mazaheri and Jensen, 2010). Thus, although frontal midline evoked responses and frontal theta oscillations are frequently reported together, they represent different neural processes and likely reflect changes across different frequencies. It has been demonstrated that most of the mid-frontal signal that is relevant for cognitive control is contained within ongoing theta oscillations, and not the evoked signal (Cohen and Donner, 2013; Gartner et al., 2015), and that theta oscillations play a much more important functional role than evoked responses (e.g., Jensen and Colgin, 2007). Therefore, the focus of the current study is on frontal theta oscillations with the phase-locked evoked activity removed.

Although frontal theta oscillations are hypothesised to be a mechanism for cognitive control, it is not yet clear whether theta plays a functional role in this process or is alternatively a generic alarm signalling only the need for control (Cavanagh and Frank, 2014). Frontal theta oscillations have been associated with a variety of control tasks, including working memory (Jensen and Tesche, 2002), mental arithmetic (Gartner et al., 2015), as well as response preparation and post-error activity (Womelsdorf et al., 2010). Theta is also related to behavioural outcomes, suggesting there is a connection between the motor cortex and features of frontal theta such as latency and amplitude. For example, frontal theta was decreased and with shorter latency to peak on trials with faster, more automatic inhibit-and-switch trials in a speeded Go/Switch task (Cheyne et al., 2012). The relative ubiquity and sensitivity of frontal theta during cognitive processing suggests it is functional and not merely contingent. However, should frontal theta be functional, the mechanism through which it exerts control over behaviour remains unknown.

One possible mechanism for frontal control of behaviour is long-range signalling via theta-gamma (60–90 Hz) phase-amplitude coupling. It has been suggested that the function of theta oscillations is to integrate information throughout the brain to impact behaviour by shaping the amplitude of local high frequency gamma activity, which may be nested within certain phases of the theta cycle (Canolty et al., 2006). In a previous study we demonstrated that gamma activity within the sensorimotor cortex is sensitive to cognitive parameters, suggesting a role for integrating cognitive information with motor signals prior to motor output (Isabella et al., 2015). However, no phase-amplitude coupling was found. Despite the widespread evidence for the existence of interregional theta coupling (Jensen and Colgin, 2007; Mazaheri et al., 2009; Polania et al., 2012; Lisman and Jensen, 2013), there is as of yet no direct evidence to support a positive relationship between frontal theta and the sensorimotor cortex. Notably, an instance of a negative correlation has been reported (Spooner et al., 2019). Thus, a mechanism through which frontal theta may exert cognitive control, if any such exists, remains to be determined.

Although there is no evidence to support a role for theta synchronisation with motor cortex activity, there is some evidence that frontal theta amplitude is sensitive to variations in cognitive processing. It has been demonstrated in a working memory task that frontal theta amplitude is sensitive to memory load (e.g., the number of items to be retained; Jensen and Tesche, 2002). Furthermore, a previous study from our group demonstrated increased error-related frontal theta amplitude across two inhibition tasks, in the absence of any behavioural differences (Isabella et al., 2015). We hypothesised that this difference in amplitude may have been related to covert differences in internal inhibitory processing between tasks.

The objective of the current study was to determine whether frontal theta is related in some way to motor signals in a behavioural control task, and whether the strength of this relationship is predictive of cognitive processing load, demonstrating a functional role in the control of behaviour rather than merely an alarm signalling the need for control, or reflecting some other cognitive function. We hypothesised that frontal theta amplitude would be sensitive to variations in cognitive load, and that increased signalling would constitute theta phase coupling with high gamma (60–90 Hz) amplitude within the sensorimotor cortex. In order to establish variations in processing load in a behavioural control task, we used a recently developed implicit stimulus pattern learning Go/Switch task (Isabella et al., 2019) paired with pupillometry as an independent measure of cognitive effort (that is, the amount of effort one exerts to process a given load) (van der Wel and van Steenbergen, 2018). Sixteen healthy adults performed this task during simultaneous pupillometric and whole-head magnetoencephalographic (MEG) recordings in order to measure oscillatory neural activity from the frontal and sensorimotor cortices.

## Materials and Methods

### Subjects

Sixteen healthy right-handed adults (8 females, range 22–31 years) participated in this experiment. All subjects were recruited from the Toronto area and provided informed consent using protocols approved by the Hospital for Sick Children Research Ethics Board. Subjects were compensated 60 CAD for their participation.

### Go/Switch Task

**FIGURE 1:**
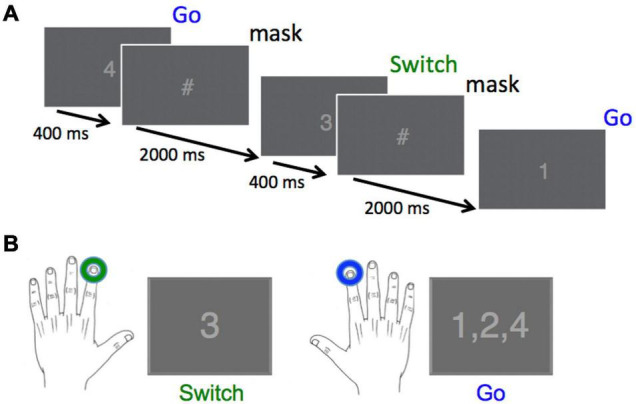
Task design and required responses. **(A)** Digits were presented every 2,400 ms for a duration of 400 ms, followed by a stimulus mask (“#”) for a duration of 2,000 ms. Overall probability for each of the four stimuli was 25%. **(B)** The correct response to the Switch stimulus (“3”) was a left index finger button press, whereas the correct response to all other stimuli was a right index finger button press (Reprinted with permission from Isabella et al., 2019).

The Go/Switch task employed in this study was similar to that used in a previous study (Isabella et al., 2019). All subjects were presented with a rapid stream of digits from “1” to “4”, where each target had an equal 25% probability of occurrence. Each stimulus was displayed for a fixed duration of 0.4 s, followed by a stimulus mask (“#”) that was displayed for an additional 2 s until the presentation of the next digit, for a total inter-trial interval of 2.4 s (Figure 1). Stimulus isoluminance was achieved by controlling the number of pixels for the stimuli and mask. The subjects were informed that they were performing a go-switch task, for which the default movement to stimuli 1, 2, or 4 was a button press with the right index finger, with instructions to switch response hands to the left index finger when presented with the target “3” stimulus. We did not counterbalance the direction of switching across hands as it has already been demonstrated that this has no effect on behavioural or neuromagnetic measures (Cheyne et al., 2012).

Subjects performed this task over 244 trials across each of 6 blocks. When time permitted, some subjects performed a 7th block. Each block began with 4 trials containing stimuli (digits 1–4) chosen at random. Subjects were uninformed that the remaining 240 stimuli were presented in 30 repeats of an 8-trial probabilistic sequence (3-1-4-3-2-4-1-2), known to induce pattern learning in adults (Isabella et al., 2019) and also in typically developing children (aged 7–12 years) during a serial reaction time task (Gabriel et al., 2011). Stimuli for 90% of trials followed the sequence order (Pattern), whereas for the remaining 10% of trials, the required response for the individual trial within the 8-trial sequence did not follow the sequence order (Deviant).

Task instructions were to “respond as quickly as possible without committing too many errors.” They were informed that they would receive a $2 performance reward for each block with overall RT under 0.4 s and error rate under 30% for right (Go) and left (Switch) responses. Once the target RT and error rate were achieved, subjects were instructed to maintain or improve their scores in order to continue receiving the $2 monetary reward for each block (for a maximum reward of $12), in order to maintain motivation for the duration of the experiment. Before commencing the test blocks, subjects performed a practice block consisting of the same stimuli presented in random order, for 64 trials lasting 2.5 min. For the practice block only, feedback in the form of RT was presented on the left side of the screen after each response to help subjects determine their speed-accuracy strategy. Ahead of the test blocks, it was suggested to use the timing of the stimulus mask at 0.4 s as a benchmark for their RT on each trial.

Subjects were told that the goal of the study was to test the effect of a particular strategy, that was to covertly say the number as it appears on the screen. Following task performance on each of the 6 blocks, subjects were presented with the average reaction times and error rates for Go and Switch responses and were encouraged to improve their score for the next block. At the end of the experiment, subjects were asked for general feedback on the task, and to write out a sample stream of stimuli from the experiment. This was in order to assess whether the presence of the sequence of stimuli was explicitly learned.

### Recordings

Neuromagnetic activity was recorded using a whole head 151-channel CTF MEG system (MISL, Coquitlam, BC, Canada) in a magnetically shielded room. Data were collected at a rate of 600 samples/s. T1-weighted structural MR images were obtained from each subject using a Siemens 3T Magnetom Trio scanner. Small coils placed at fiducial locations (nasion and preauricular points) were used to monitor head position during recording and co-register source images to the subject’s MRI. Subjects sat upright in an adjustable chair and responses were collected using a nonmagnetic fiber optic response pad (LUMItouch Response System, Lightwave Medical Industries, Burnaby, Canada). Stimuli were presented using Presentation Software (version 14.9)^1^ via a LCD projector on a back-projection screen.

Real-time task-evoked pupil responses were measured using an EyeLink 1000 system (SR Research, Ottawa, Canada), recording at 600 Hz and synchronised with the neuromagnetic activity. Pupil diameter (PD) was measured in arbitrary units.

### Analysis

#### Behavioural Analysis

##### Response Types

Response types were defined as follows:

• Pattern Go (PGo): correct Go response (right index) to the Go stimulus (the digits 1, 2, or 4) matching the repeated pattern, i.e., occurring in the expected location within the sequence.
• Pattern Switch (PSw): correct switch response (left index) to the probable Switch stimulus (the digit 3) matching the repeated pattern.
• Deviant Go (DGo): correct go response (right index) to a Go stimulus (digit 1, 2, or 4) deviating from the repeated pattern, i.e., a Go stimulus where an expected “3” stimulus would have occurred requiring a Switch to a left index response. For example, given the intact pattern (3-1-4-3-2-4-1-2), a sample stream of stimuli with a Deviant Go trial (underlined) would be: 3-1-4-1-2-4-1-2. If the Pattern stimulus was expected, a change from a Switch to a Go response was required.
• Deviant Switch (DSw): correct switch response (left index) to improbable switch “3” stimulus, deviating from the repeated sequence, i.e., where the expected Pattern stimulus would have required a Go response. For example, a sample stream of stimuli with a Deviant Switch trial (underlined) would be: 3-1-4-3-2-3-1-2. For this response type, if the Pattern stimulus was expected, a change from a Go to a Switch response was required.

Importantly, all trial types as defined were preceded by a Pattern Go response. Any missed (nonresponse within 1.5 s of stimulus presentation) trials were rare and not included in any analyses. All trials following a “3” stimulus were not included in the analysis, as subjects quickly learned that the Switch stimulus “3” never occurred twice in succession and therefore could explicitly predict that a Go trial would follow a Switch trial. Any trials containing the incorrect response were also not included in the analysis. Therefore, all trials included in the analysis were preceded by a correct Go response. Furthermore, as differences were observed between pattern and deviant trials from the first block, blocks 2–6 were included for analysis for Pattern trials (i.e., upon learning the pattern), and blocks 1–6 for Deviant trials.

##### Reaction Times

RT was measured as the difference in time between stimulus onset and the button press within 1.5 s of each trial. Any trials that did not include a physical button press within that time frame or contained more than one button press were not included in the analyses.

#### Pupil Diameter

Continuously recorded pupil diameter (PD) data was segmented into epochs and time-locked to stimulus onset. Eye blinks were linearly interpolated using a custom Matlab script (The MathWorks, Inc., Natick, MA, United States), low pass filtered at 4 Hz, and then z-transformed within participants to minimize inter-subject variability, as performed by Smallwood et al. (2011). Importantly, these preprocessing steps are in line with those outlined by Kret and Sjak-Shie (2019).

Pre-stimulus pupil activity was measured as the mean z-scored pupil diameter for the 0.4 s preceding stimulus onset, which was then subtracted from the entire trial. Mean task-evoked pupil responses were measured as the mean z-scored PD for the 2.0 s following stimulus onset (until the subsequent pre-stimulus time period).

#### Magnetoencephalographic Analysis

Continuously recorded MEG data were segmented into epochs centered upon the button response (response-locked) for each of the four response types and included blocks described above. Localization of brain activity was carried out using frequency-based beamformer algorithms, using a single-sphere head model (Lalancette et al., 2011) implemented in the BrainWave Matlab toolbox developed at the Hospital for Sick Children (Jobst et al., 2018). Continuous head localization was used to monitor head motion throughout the recordings and trials were rejected off-line if head motion exceeded 5 mm.

We measured changes in induced cortical oscillations using the synthetic aperture magnetometry (SAM) algorithm (Robinson and Vrba, 1999) in the frontal and sensorimotor cortices, in the theta, beta and high gamma frequency bands. Spatial normalisation of volumetric source images (4 mm resolution) to the MNI (T1) template brain was carried out using SPM8 (Wellcome Centre for Human Neuroimaging, London, United Kingdom). Talairach coordinates of peak activations were determined from the normalised images using the MNI to Talairach daemon (Lancaster et al., 2000). Group beamformer source images of the peak activations were superimposed onto a 3D rendered image of Colin-27 (CH2.nii) average brain using BrainWave and thresholded for illustration purposes only.

In order to account for different numbers of trials for each response type, a separate dataset was created that included equal numbers of all 4 trial types of interest (selected at random) to calculate common beamformer weights. These weights were used to compute whole-brain pseudo-t difference images for individual trial types by subtracting the source power during an active time window of 500 ms duration from a prestimulus baseline period of equal duration in the theta frequency band (4–8 Hz), 300 ms duration in the beta frequency band (15–30 Hz) or 200 ms duration in the high gamma (60–90 Hz) frequency bands. The sizes of these time windows were chosen in order to capture a minimum of 2–4 cycles of the lowest frequency of interest within a given window. The baseline time windows were set from 1.1 to 0.6 s preceding movement onset for the theta frequency band, 0.8 to 0.5 s preceding movement onset for the beta frequency band, and 0.6 to 0.4 s preceding movement onset for the gamma frequency band, in order to obtain a stable baseline for each. These baseline time windows were chosen based on empirical investigation of the data, in order to ensure the baseline begins after activity from the previous trial had terminated, and before activity in the current trial began (Gross et al., 2012). This empirically-determined backward shift for lower frequency bands inherently accounts for the time-frequency resolution tradeoff that accompanies lower frequency analysis. The active time window was shifted in 50 ms increments from the period immediately following the baseline window, up to 0.5 s following the button-press response. In order to determine the source of the task-induced oscillatory activity within the frequency band of interest, images were searched for activation within the time window of interest.

To address our hypothesis of comparing task-induced source activity across trial types, time-frequency representations (TFRs) were constructed from source waveforms at the peak locations determined above within the pseudo-t images. This was accomplished using a Morlet wavelet frequency transformation (Tallon-Baudry et al., 1997) of single trial source activity in 1 Hz steps using the following formula:


$$w\left(t,f_{0}\right)=A\exp\left(-t^{2}/2\sigma_{t}^{2}\right)\exp\left(2i\pi f_{0}t\right)$$

Wavelets are normalised so that their total energy is 1, using the normalisation factor A that is equal to:


$${\left(\sigma_{t}\sqrt{\pi }\right)}^{-1/2}$$

A convolution of the complex wavelet with the MEG signal is then derived and the magnitude of this convolution used to create each TFR for each trial type of interest. This value is then converted to percent change in power relative to the same pre-movement baseline. Importantly, in order to exclude evoked activity, mean power was subtracted from single trial power, within-subject for each condition, to remove phase-locked activity and image primarily induced oscillatory activity (Keil et al., 2010). Mean power was then calculated for each subject and condition over the time window of interest.

### Statistical Analyses

RT was log-transformed to normalize its distribution. To examine differences between trial types across RT, PD, and mean oscillatory power, 2-by-2 within-subject repeated measures ANOVAs were conducted (factors were Sw and pattern), and partial eta squared (η_*p*_^2^) was used to calculate effect sizes. Post-hoc comparisons were conducted using t-tests between PGo and PSw, PGo and DGo, and PSw and DSw, resulting in Holm ranked significance levels of 0.017, 0.025, and 0.05 (ranked from lowest p-value to highest). Thus, the null hypothesis is rejected for post-hoc t-tests only if the p-value of the test is less than its rank-order significance level. Other corrections for multiple comparisons were similarly performed with Holm-adjusted values.

In order to investigate relationships between frontal theta and the other outcome measures of interest (RT, PD, beta, gamma), a regression approach was used in order to control for possible interactions of task variables (Sw and pattern). Relationships between measures were determined using the sum of squares from a repeated measures ANOVA between variables according to the following formula:


$$r=\sqrt{\frac{SSpvs}{SSpvs+SSr}}$$

where SS*pvs* is the sum of squares for the predictor variables (as determined by the regression), SS*r* is the sum of squares for the residual, and *r* is the correlation coefficient (Bland and Altman, 1995). All statistical tests were performed using R (R Core Team, 2017).

### Phase and Amplitude Analyses

The time-series for sensorimotor gamma and frontal theta were further analysed for phase-amplitude coupling, as well as phase synchrony between theta sources. Similar to several recent studies, cross-frequency phase-amplitude coupling was analysed using phase-locking value (PLV, also known as synchronisation index or phase locking modulation index) and phase synchrony was analysed using debiased weighted phase lag index (dwPLI) (Hebscher et al., 2019; Siebenhühner et al., 2020). Both PLV and dwPLI are computed as the vector length of phase differences over time, such that larger values reflect less variability in phase differences between two signals. The following analyses were performed using custom-written Matlab code (PLV) or code from the fieldtrip toolbox^2^ (Oostenveld et al., 2011) in combination with custom-written Matlab code (dwPLI).

For PLV we followed the methods outlined by Cohen (2008), where phase and amplitude of the single-trial band-filtered signals were extracted from the Hilbert transform, after subtracting the trial-locked average to remove phase-locked activity. The synchronisation between gamma and theta time series was calculated over baseline and 3 sliding active windows of 0.5 s duration (−0.5 to 0.5 s) using PLV:


$$PLV=\frac{1}{N}\sum_{t=1}^{N}e^{i}\left|\phi_{lt}-\phi_{ut}\right|$$

where ϕ_*lt*_ is the phase value of the theta time series at time *t*, and ϕ_*ut*_ is the phase value of the gamma amplitude envelope filtered at the lower frequency (4–8 Hz) for time window *t* (Siebenhühner et al., 2020). Statistical analysis proceeded by 1,000 random permutations of trial indices for gamma amplitude over a randomly selected 0.5 s window within the active time period (−0.5 to 0.5 s). A *p* < 0.05 statistical threshold for PLV was set at the 95th percentile of the distribution of mean values for the random permutations.

For dwPLI we followed the methods outlined by Vinck et al., 2011 and implemented by Babapoor-Farrokhran et al., 2017, where the imaginary component of the cross-spectrum of each theta source pair was calculated over baseline and 3 sliding active windows of 0.5 s duration (−0.5 to 0.5 s) as follows:


$$dwPLI=wPLI^{2}={\left(\frac{\sum_{j=1}^{N}ℑ\left\{X_{j}\right\}}{\sum_{j=1}^{N}\left|ℑ\left\{X_{j}\right\}\right|}\right)}^{2}$$

where ℑ{*X*_*j*_} is the imaginary component of the cross-spectrum of a given time window in the *j*th trial. dwPLI was preferred for this analysis comparing task effects across different trial numbers as it has negligible sample size bias for small data sets (Vinck et al., 2011). As above, statistical analysis proceeded by 1000 random permutations of trial indices for theta phase. A *p* < 0.05 statistical threshold for dwPLI was set at the 95th percentile of the distribution of mean values for the random permutations.

For each comparison where dwPLI exceeded the statistical threshold, the active window with the greatest increase over baseline in dwPLI was selected for analysis of task effects using a 2-by-2 within-subject repeated measures ANOVAs were conducted (factors were Switch and pattern), as above. P-values were Holm-adjusted for the number of comparisons (5 theta sources resulted in 10 comparisons between each source). Thus, the null hypothesis was rejected for ANOVAs only if the *p*-value of the test was less than rank-order significance level.

## Results

### Behavioural Results

All 16 subjects complied with task instructions, completed a minimum of 6 blocks and provided feedback on the task. All subjects earned the maximum $12 performance reward, in addition to the $60 compensation for participation. As per our previous study (Isabella et al., 2019), none of the subjects were able to replicate the stimulus sequence at the end of the experiment and failed to provide any evidence of explicit knowledge of the stimulus sequences. The mean number of trials of each trial type were as follows: PGo = 563.6, DGo = 36.8, PSw = 237.5, DSw = 31.8. Error trials and any trial following a Switch stimulus were not analysed for this study.

#### Reaction Times

**FIGURE 2:**
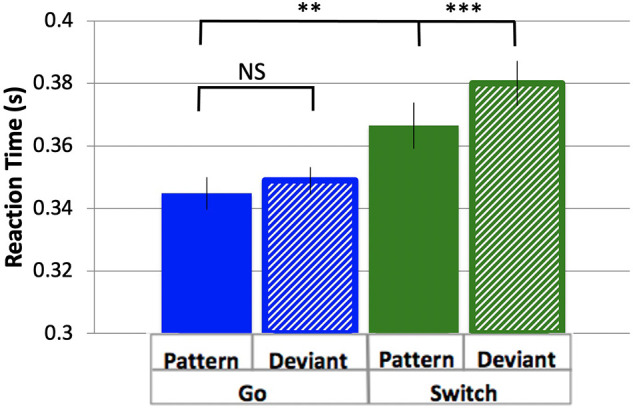
Reaction time. Mean RTs and standard errors for response types Pattern and Deviant Go, Pattern and Deviant Switch. Pattern Go, Pattern Switch and Deviant Switch response types were all significantly different from each other (all *p* < 0.003), but not Pattern and Deviant Go (*p* = 0.30). ^**^p < 0.01, ^***^p < 0.001 for post-hoc t-tests of PGo vs PSw, and PSw vs DSw.

To determine the effects of task (Switch = Go/Sw and Pattern = Pattern/Deviant) on responses, reaction times were measured as the time between stimulus onset and the button press response for the four trial types of interest, in order of decreasing probability: PGo, DGo, PSw, and DSw. The overall pattern of results replicates our previous findings (Isabella et al., 2019), with differences between Pattern and Deviant trials that were evident from the first block (DSw > PSw, *p* = 0.03), demonstrating rapid learning of the stimulus pattern. Since there was evidence of pattern learning from the first block, all analysis of Pattern trials contained data from blocks 2–6 only, thus excluding the first block during which the pattern was learned. Mean RT was greater for Sw responses over Go, and greater for Deviant over Pattern trials (mean PGo = 0.345 s, DGo = 0.349 s, PSw = 0.366 s, DSw = 0.380 s, Figure 2). To determine the effects of task parameters on reaction times, a 2-way ANOVA was conducted on log-transformed averaged reaction times, revealing a statistical main effect of Switch [*F*(1,15) = 16.55, *p* = 0.001, η_*p*_^2^ = 0.52] and of pattern [*F*(1,15) = 10.17, *p* = 0.006, η_*p*_^2^ = 0.40], and no interaction between the two [*F*(1,15) = 4.29, *p* = 0.06]. Post-hoc comparisons revealed significant differences between PGo and PSw, as well as PSw and DSw (all *p* < 0.003), but not PGo and DGo (*p* = 0.13). Switch responses of all types were delayed, but Deviant trials were delayed only for Sw trials and not Go trials (i.e., DGo). These results demonstrate an inverse relationship between response duration and variations in stimulus probability for all trial types except for DGo.

### Physiological Measures

#### Task-Evoked Pupil Responses

**FIGURE 3:**
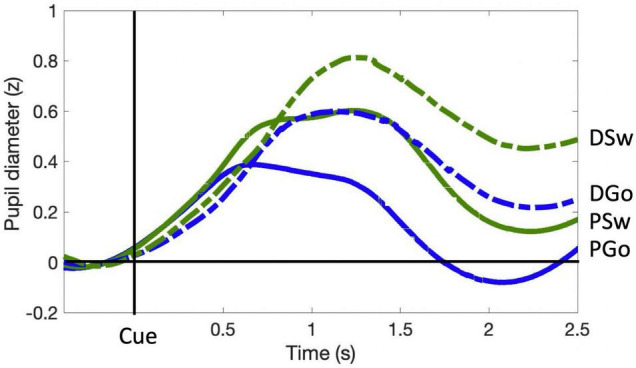
Pupil diameter time course. Pupil diameter is within-subject z-scored, and time-locked to cue onset. Pre-Stimulus PD for each trial was calculated as the average z-score value within the 0.4 s preceding stimulus onset, and was subtracted from the entire trial. Mean task-evoked pupil response for each trial type was calculated as the average z-score value over 2.0 s following stimulus presentation.

**FIGURE 4:**
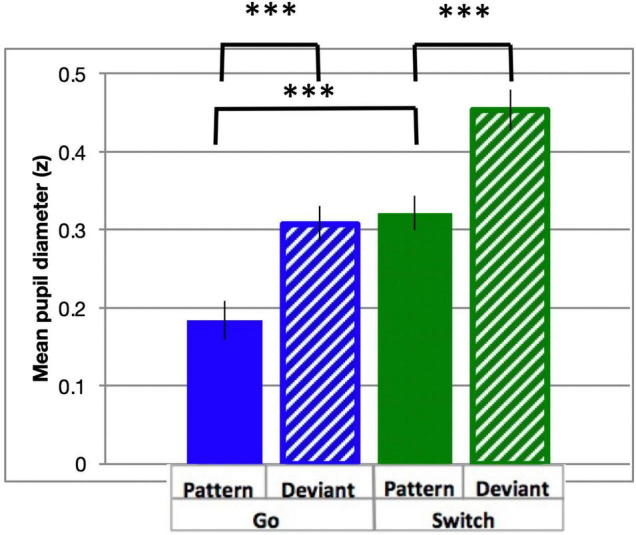
Task-evoked pupil diameter. Mean PD (in z-scores) and standard errors for all response types across the active window (0–0.2 s from cue onset). All response types were significantly different (all *p* < 0.001). ^***^p < 0.001 for post-hoc *t*-tests of PGo vs DGo and PSw, and PSw vs DSw.

Pupil dilation is a well-established covert measure of quantifying cognitive control (Kahneman and Beatty, 1966). In the current study, similar to RT, all analysis of Pattern trials contained data from blocks 2–6 only, excluding the first block during which the pattern was learned. PD followed a typical time course, beginning at a minimum prior to stimulus onset, and dilating to a maximum diameter within 0.5 to 1.5 s (Figure 3). Diameters generally returned to approximately pre-stimulus levels following PGo trials ahead of the next trial at 2.4 s (n.b. all trials included in the analysis were preceded by PGo trials). PD was calculated as the mean baselined z-scored pupil diameter for 2 s following stimulus onset (time 0 to 2 s), and was smallest for PGo trials, and increased for each of DGo, PSw and DSw trials (mean PGo = 0.25 ± 0.04 z, DGo = 0.44 ± 0.03 z, PSw = 0.46 ± 0.03 z, DSw = 0.56 ± 0.03 z; Figure 4). In order to determine the effects of the task parameters on PD, a 2-way ANOVA was conducted, revealing a statistical main effect of Switch [*F*(1,15) = 176.2, *p* < 0.001, η_*p*_^2^ = 0.92] and of pattern [*F*(1,15) = 76.64, *p* < 0.001, η_*p*_^2^ = 0.84], and no interaction between the two (*F*(1,15) = 3.46, *p* = 0.08). Post-hoc comparisons revealed significant differences between PGo and PSw, PGo and DGo, and Pattern and Deviant Sw (all *p* < 0.001). These results reveal a parametric increase in PD with decreasing stimulus probability, and contrasted with RT results, consistent with previous findings that PD and RT index different processes within cognitive control (Isabella et al., 2019).

### Neuromagnetic Measures

#### Frontal Theta

**FIGURE 5:**
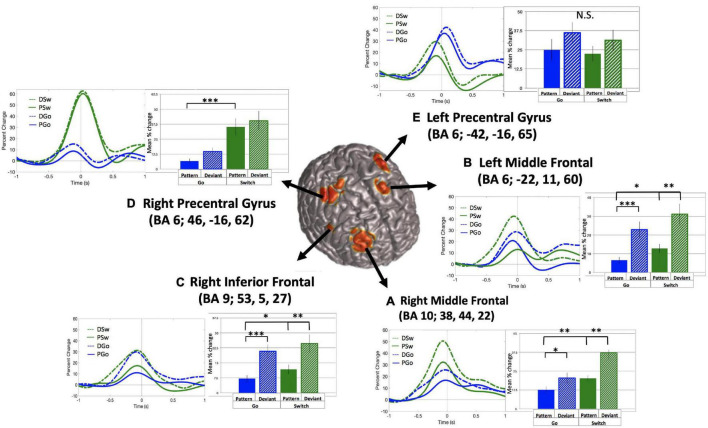
Frontal theta oscillatory power. Source localization for 5 frontal theta sources **(A–E)**, with baseline set to –1.1 to –0.6 s relative to response onset. Time courses and mean theta power (in percent change over baseline) and standard errors are presented for all trial types for each source. *p < 0.05, ^**^p < 0.01, ^***^p < 0.001 for post-hoc *t*-tests of PGo vs DGo and PSw, and PSw vs DSw.

The relationship between variations in cognitive control and frontal theta oscillations was of critical interest in the current study. SAM beamformer analysis revealed consistent theta band (4–8 Hz) oscillatory activity in 5 frontal sources: (A) Right middle frontal gyrus (mean Talairach coordinates: x = 38, y = 44, z = 22, BA 10), (B) Left middle frontal gyrus (mean Talairach coordinates: x = −22, y = 11, z = 60, BA 6), (C) Right inferior frontal gyrus (mean Talairach coordinates: x = 53, y = 5, z = 27, BA 9), (D) Right precentral gyrus (or premotor cortex, mean Talairach coordinates: x = 46, y = −16, z = 62, BA 6), and (E) Left precentral gyrus (or premotor cortex, mean Talairach coordinates: x = −42, y = −16, z = 65, BA 6), as depicted in Figure 5 (baseline = −1.1 to −0.6 s).

For all sources, theta power followed a typical time course, increasing to a maximum just prior to the response. Mean theta power was calculated as the mean percent change in power from 0.4 s prior to until 0.2 s after the button press response, relative to the pre-stimulus baseline. Across sources, theta power was smallest for PGo trials, and generally increased across task parameters, although relative weight of task effects and statistical significance varied across regions. Mean values and ANOVA results for all five frontal sources are presented in Table 1. Interestingly, among all theta sources, only the Right Middle Frontal showed significant effects of both task parameters, Switch and pattern. This result supports our hypothesis that frontal theta is sensitive to parametric increases in cognitive control.

**TABLE 1:** Frontal theta results.

					Main effects		Interactions
Source	Mean PGo	Mean DGo	Mean PSw	Mean DSw	Switch	Pattern	Switch*Pattern
A: R Middle Frontal	12.55 +/− 2.22	20.46 +/− 13.13	20.27 +/− 1.86	37.15 +/− 4.49	F(1,15) = 17.61, ***p < 0.001, η_*p*_^2^ = 0.54	F(1,15) = 20.46, ***p < 0.001, η_*p*_^2^ = 0.58	n/s
B: L Middle Frontal	6.54 +/− 1.74	22.87 +/− 4.33	12.76 +/− 2.47	31.13 +/− 5.56	F(1,15) = 4.40, *p* = 0.053, η_*p*_^2^ = 0.23	F(1,15) = 46.91, ***p < 0.001, η_*p*_^2^ = 0.76	n/s
C: R Inferior Frontal	7.11 +/− 1.70	20.80 +/− 3.39	11.77 +/− 2.34	24.73 +/− 4.56	F(1,15) = 1.56, *p* = 0.23, η_*p*_^2^ = 0.09	F(1,15) = 32.55, ***p < 0.001, η_*p*_^2^ = 0.68	n/s
D: R Precentral	6.71 +/− 1.91	14.67 +/− 3.16	35.26 +/− 7.06	40.36 +/− 8.07	F(1,15) = 21.52, ***p < 0.001, η_*p*_^2^ = 0.59	F(1,15) = 7.52, *p* = 0.015, η_*p*_^2^ = 0.33	n/s
E: L Precentral	24.96 +/− 6.98	36.07 +/− 6.87	22.47 +/− 5.01	31.21 +/− 6.71	F(1,15) = 7.40, *p* = 0.016, η_*p*_^2^ = 0.33	F(1,15) = 12.08, ***p = 0.003, η_*p*_^2^ = 0.45	n/s

*Mean theta power in percent change over baseline (+/− standard error) are presented for each trial type and frontal theta source. ANOVAs were calculated within subject across repeated measures of Pattern and Switch. Only main effects are presented as no significant interactions were found. Significant results are highlighted in yellow (for 5 Holm-adjusted rank-order significance levels).*

#### Sensorimotor Beta

**FIGURE 6:**
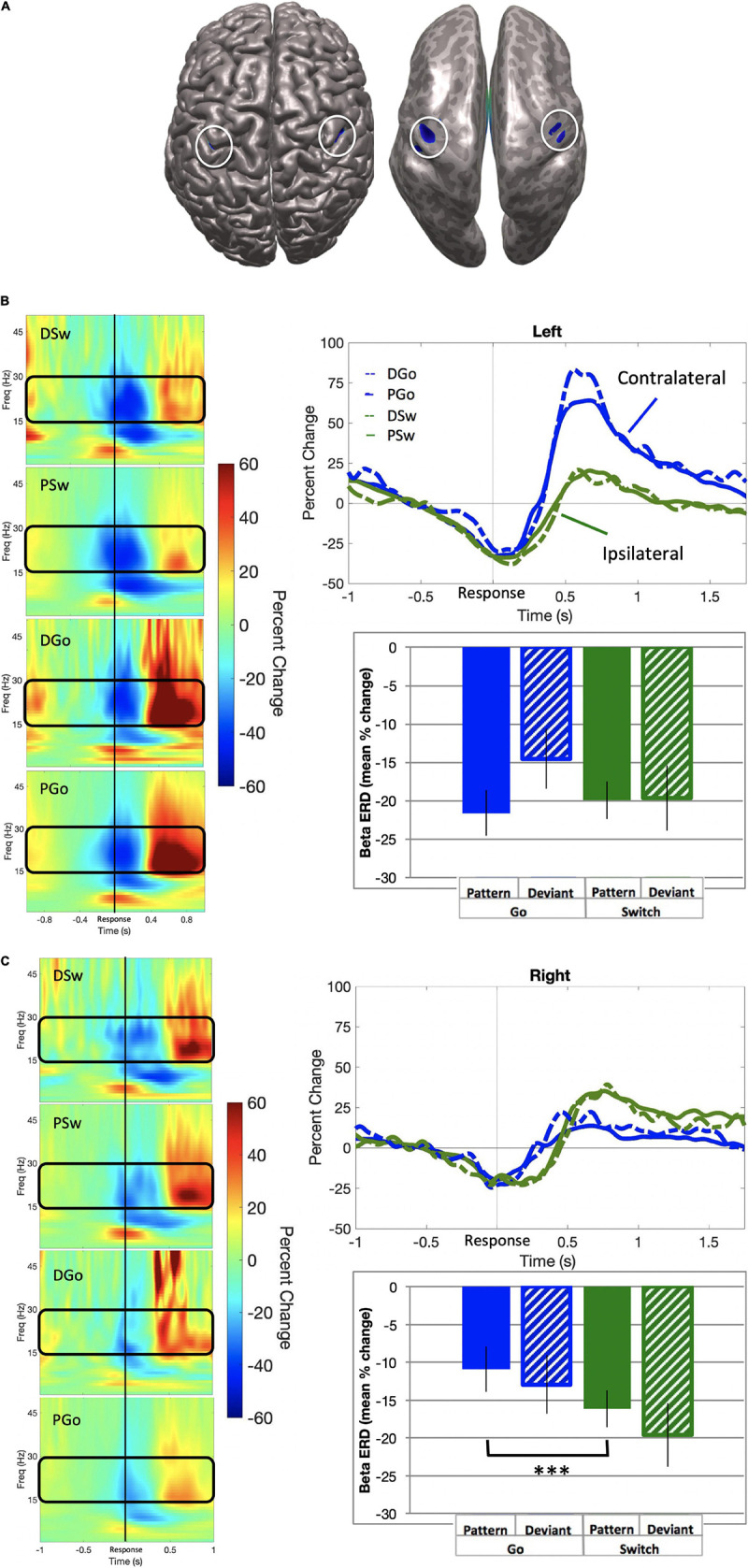
Beta event-related desynchronisation. **(A)** Source localization in left (*x* = –38, *y* = –21, *z* = 43; BA 3) and right (*x* = 38, *y* = –21, *z* = 40; BA 3) motor cortex, also shown on an inflated brain (right), with baseline set to –0.7 to –0.4 s relative to response onset. **(B)** Left motor cortex. (Left panel) Time-frequency representations for all trial types, with the frequency bin of interest (15–30 Hz) outlined in black. (Top panel) Time courses for beta ERD in the left motor cortex, for all trial types. (Bottom panel) Mean beta ERD (in percent change over baseline) and standard errors over 150 ms preceding response onset for all trial types. No significant differences were found. **(C)** Right motor cortex. (Left panel) Time-frequency representations for all trial types, with the frequency bin of interest (15–30 Hz) outlined in black. (Top panel) Time courses for beta ERD in the left motor cortex, for all trial types. (Bottom panel) Mean beta ERD (in percent change over baseline) and standard errors over 150 ms preceding response onset for all trial types. Post-hoc analysis revealed a significant increase for Pattern Switch trials over Pattern Go trials (*p* < 0.001), but not Deviant Go trials (*p* = 0.49). ^***^p < 0.001 for post-hoc t-tests of PGo vs PSw.

Beta event-related desynchronisation (ERD) is characterised by decreasing power preceding responses, but less so during uncertainty, and may be causally relevant to response execution. SAM beamformer analysis revealed consistent beta ERD oscillatory activity bilaterally in the sensorimotor cortex (mean Talairach coordinates left: x = −38, y = −21, z = 43; right: x = 38, y = −21, z = 40) for correct P and DGo and Sw trials, prior to responding (Figure 6; baseline = −0.7 to −0.4 s). We calculated the mean beta ERD power from 0.4 s preceding response onset until subsequent return to baseline. Mean power was calculated separately for each hemisphere. In the left motor cortex, beta power decreased for all trial types (mean PGo = −21.6 ± 3.0%, DGo = −14.5 ± 3.9%, PSw = −19.9 ± 2.4%, DSw = −19.6 ± 4.2%, Figure 6B bottom panel). In the right motor cortex, similar beta power decreases were observed for all trial types (mean PGo = −10.9 ± 2.0%, DGo = −12.9 ± 2.6%, PSw = −16.1 ± 2.7%, DSw = −19.6 ± 2.3%, Figure 6C bottom panel).

In order to determine whether there were any effects of task parameters on pre-response beta activity, a 2-way ANOVA was conducted on all four trial types of interest for mean power in the left motor cortex, with no significant effects of Switch [*F*(1,15) = 3.93, *p* = 0.07] or pattern [*F*(1,15) = 0.0.612, *p* = 0.45]. In contrast, there was a significant effect of Switch in the right motor cortex [Switch *F*(1,15) = 16.27, *p* = 0.001, η_*p*_^2^ = 0.52; pattern *F*(1,15) = 4.42, *p* = 0.05]. Post-hoc analysis for the right motor cortex revealed statistical differences between PGo and PSw responses (*p* < 0.001), but not PGo and DGo (*p* = 0.17) or PSw and DSw responses (*p* = 0.03). These results demonstrate that left motor cortical oscillatory activity was similar across all trials types, but right motor cortical oscillatory activity was different between PGo and Sw trials. These results suggest that subjects maintained preparation of the prepotent Go response when a Switch trial was anticipated. Furthermore, parallel activation of both responses may reflect a higher cost of inhibiting the prepared Go response over delayed activation of the Switch response.

#### Sensorimotor Gamma

**FIGURE 7:**
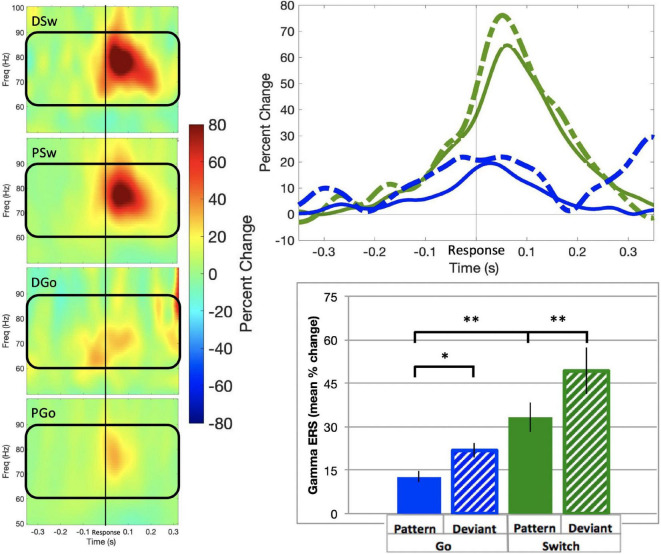
Gamma event-related synchronisation. (Left panel) Time-frequency representations of contralateral motor cortex for all trial types, with the frequency bin of interest (60–90 Hz) outlined in black. (Top panel) Time courses for gamma ERS in the contralateral motor cortex, for all trial types. (Bottom panel) Mean gamma ERS (in percent change over baseline) and standard errors preceding response onset for all trial types. All response types were significantly different. * all *p* 0.015 for post-hoc t-tests of PGo vs DGo and PSw, and PSw vs DSw.

Gamma event-related synchronisation (ERS) in the sensorimotor cortex has only recently been shown to vary with task parameters, and may be related to resolving response conflict. SAM beamformer analysis revealed consistent gamma ERS activity the sensorimotor cortex contralateral to the response hand in the same sources as beta ERD activity (mean Talairach coordinates left: x = −38, y = −21, z = 43; right: x = 38, y = −21, z = 40) for all trial types of interest, commencing approximately 200 ms prior to responding (Figure 7; baseline = −0.6 to –0.4 s). In the ipsilateral motor cortex, there was no significant gamma activity found (data not shown). We calculated the mean gamma ERS power in the contralateral motor cortex. Gamma ERS showed a similar pattern of effects as frontal theta, with the smallest ERS for PGo trials, and increasing for DGo, PSw, and DSw, respectively (mean PGo = 12.7 ± 2.0 %, DGo = 21.8 ± 2.6, PSw = 33.3 ± 5.3%, DSw = 49.3 ± 7.9 %). In order to determine the effects of task parameters on gamma activity, a 2-way ANOVA was conducted on mean power in the contralateral motor cortex, with significant effects of Switch [*F*(1,15) = 13.02, *p* = 0.002, η_*p*_^2^ = 0.46) and pattern (*F*(1,15) = 8.027, *p* = 0.013, η_*p*_^2^ = 0.35]. Post-hoc comparisons revealed differences between PGo and PSw (*p* = 0.001), between PGo and DGo (*p* = 0.015), and PSw and DSw (*p* = 0.002). This finding that, like frontal theta, sensorimotor gamma parametrically increases with decreasing stimulus probability is in line with our previous findings (Isabella et al., 2015), and suggests that sensorimotor gamma is sensitive to cognitive control parameters. Given the similarity to frontal theta, sensorimotor gamma may be involved in integrating cognitive control signals into the motor cortex.

#### Regression Analyses

**FIGURE 8:**
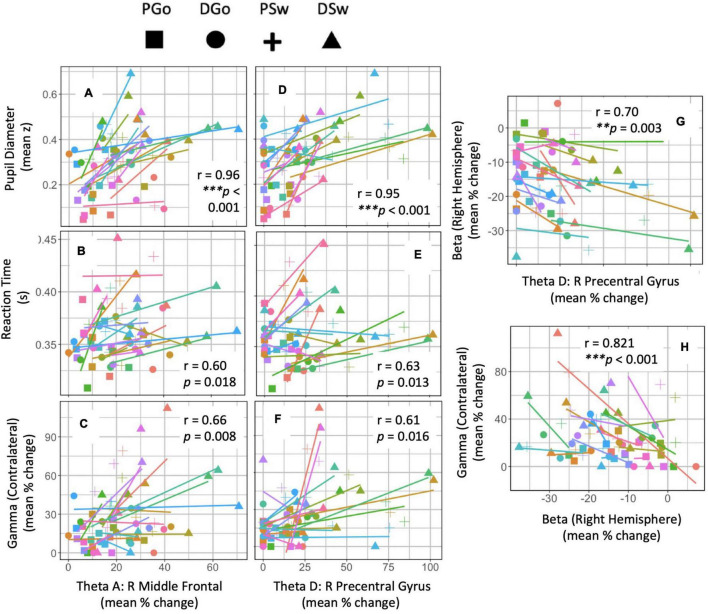
Regression analysis. Anova tables were calculated within subject across repeated measures of Pattern and Switch. The sum of squares were analysed to determine the strength (*r*) and significance (*p*) of covariance between frontal theta sources and other outcome measures of interest. **(A–H)** Data points are colour-coded for each subject, and symbols represent each trial type. * denotes significant *p*-values after Holm adjustment.

In order to investigate the relationship between frontal theta and the variety of measures in this study related to cognitive and motor control, we performed a multiple regression and analysed the sum of squares to determine the strength and significance between the measures and within subjects. Equations and results are shown in Table 2 and correlations are shown for Right Middle Frontal theta and Right Premotor theta in Figure 8. Controlling for effects of pattern and Sw, there was a significant relationship between these two frontal theta sources and PD [R Middle Frontal theta – PD: *r* = 0.964, *F*(1,1,1,13) = 168.53, *p* < 0.001; R Precentral theta – PD: *r* = 0.955, *F*(1,1,1,13) = 133.54, *p* < 0.001]. Given that these theta sources had a similarly strong relationship with PD and therefore cognitive load, the remaining regression analyses focused on these two sources. Beyond PD, regression analyses revealed relationships between both theta sources and RT as well as gamma ERS that did not survive correction for multiple comparisons [R Middle Frontal theta– RT: *r* = 0.602, *F*(1,1,1,13) = 7.40, *p* = 0.018; R Precentral theta – RT: *r* = 0.626, *F*(1,1,1,13) = 8.36, *p* = 0.013; R Middle Frontal theta – gamma: *r* = 0.655, *F*(1,1,1,13) = 9.78, *p* = 0.008; R Premotor theta – gamma: *r* = 0.608, *F*(1,1,1,13) = 7.61, *p* = 0.016]. Interestingly, although it was hypothesised that frontal theta might relate to the lateralisation (contralateral – ipsilateral) of beta ERD (reflecting varied response preparation due to task parameters), these measures were not correlated. Instead, there was a significant intrahemispheric correlation between Right Premotor theta and right beta [*r* = 0.703, *F*(1,1,1,13) = 12.73, *p* = 0.003]. Interestingly, there was a strong bi-hemispheric correlation between beta ERD in the right hemisphere and (contralateral) gamma ERS [*r* = 0.821, *F*(1,1,1,13) = 26.96, *p* < 0.001].

**TABLE 2:** Anova tables.

Independent variable	Dependent variable	SS_*predictor*_	ss_*residuals*_	*r*	*p*	*F*
Theta A	PD	0.193	0.015	0.964	***8.09e-09	168.53
Theta D	PD	0.200	0.019	0.955	***3.28e-08	133.54
Theta A	RT	0.003	0.005	0.602	0.018	7.40
Theta D	RT	0.005	0.007	0.626	0.013	8.36
Theta A	Gamma ERS	2356	3132	0.655	0.008	9.78
Theta D	Gamma ERS	3556	6079	0.608	0.016	7.61
Theta A	Beta Laterality	217	3624	0.238	0.393	0.78
Theta D	Beta Laterality	185	2844	0.247	0.846	0.374
Theta A	Beta Right	159.28	218	0.650	0.009	9.505
Theta D	Beta Right	277.11	283	0.703	**0.003	12.734
Beta Right	Gamma ERS	7583	3656	0.821	***0.0001	26.96

*The ANOVA equation for each dependent variable was calculated, in the form: (Dependent Variable)∼[((Independent Variable) + Go + Patt) + Error (subject/((Independent Variable) + Go + Patt))]. The sum of squares for the predictors (SSpredictor) and residuals (SSr) are shown, and were used to calculate the r-values, F-statistic, and p-values for each regression. Significant results are highlighted in yellow, as in Figure 8 (for 11 Holm-adjusted rank-order significance levels).*

Taken together, these analyses reveal a significant relationship between the various frontal theta sources and PD (but especially the right middle frontal), while PD is known to have a very tight relationship to cognitive load. Furthermore, frontal theta activity in the right premotor area demonstrated a closer relationship with more motor-related measures such as RT and sensorimotor beta ERD than more anterior theta sources. These widely distributed correlations with theta suggests a very meaningful role for this frontal signal within the cognitive control of behaviour during this task.

#### Phase and Amplitude Analysis

Based on the above regression findings, phase-amplitude coupling (PAC) analysis was performed between sources. Analysis of long-range synchronisation between theta phase and sensorimotor gamma PAC revealed no significant coupling for any trial type or theta source. Mean PLV was 0.006, while the *p* = 0.05 cutoff was 0.22, and therefore was not analysed further.

**FIGURE 9:**
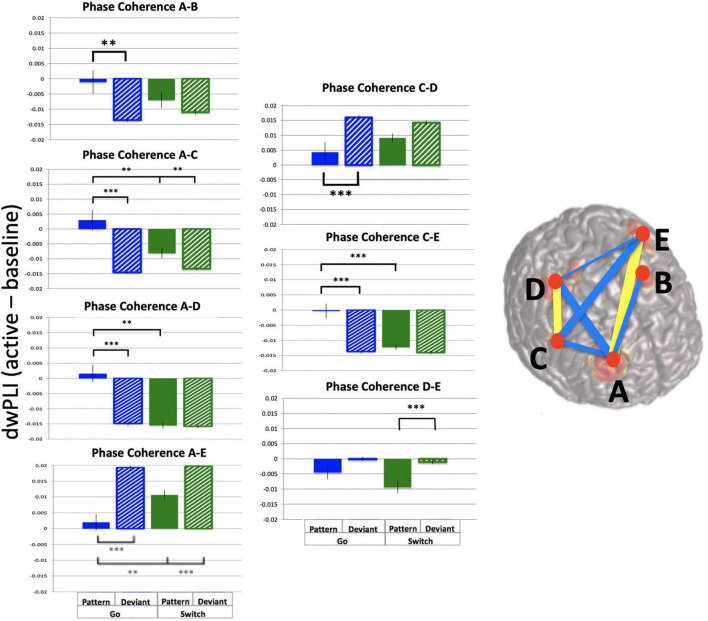
Frontal theta task-related phase coherence. Task-related theta phase coherence (using dwPLI) across 5 frontal sources [(A): Right Middle Frontal, (B): Left Middle Frontal, (C): Right Inferior Frontal, (D): Right Precentral gyrus, (E): Left Precentral gyrus). Baseline was set to –1.1 to –0.6 s relative to response onset. dwPLI during baseline and active windows was very strong (dwPLI of 0.94), there were subtle task-related changes in coherence. On the template brain blue lines represent decreases in dwPLI during the active window (–0.25 to 0.25 s relative to response onset) compared with baseline, while yellow lines represent increases during the same time period. Lines are weighted to mean task-related change in dwPLI (see Supplementary Table 1 for mean values and ANOVA results). For each significant task-related source pair, bar graphs represent mean dwPLI and standard errors across trial types. ^**^p < 0.01, ^***^p < 0.001 for post-hoc *t*-tests of PGo vs DGo and PSw, and PSw vs DSw.

In contrast, analysis of phase coherence between the 5 frontal theta sources revealed strong dwPLI across sources and trial types. Mean dwPLI values were 0.94 (baseline and active windows) while the *p* = 0.05 cutoff was 0.45. Importantly, dwPLI values exceeded threshold values for each source pair for all subjects, trial types and time points. In order to determine task effects on theta phase coherence, the active window with the largest mean dwPLI was selected (−0.25 to 0.25 s, mean change in dwPLI = 0.007). Baseline-subtracted dwPLI values were used for ten 2-way ANOVAs of across trial types, one for each source pair. Results are illustrated in Figure 9 (summary table is available in Supplementary Material). Source pairs that showed task-related increases in dwPLI are denoted in yellow, and task-related decreases in dwPLI are denoted in blue. Most source pairs had a main effect of pattern, except for most connections with Left Middle Frontal. Furthermore, two source pairs had effects of both task parameters (Switch and pattern) plus interaction: Right Middle Frontal – Right Premotor pair and Right Inferior Frontal – Left Premotor pair.

Taken together, these findings demonstrate robust ongoing phase coherence between all five frontal theta sources (mean dwPLI of 0.94), with a marginal change (increase or decrease) during task performance (mean dwPLI change of 0.007).

## Discussion

In the current study we sought to test the functional role of frontal theta in cognitive control by examining its sensitivity to experimentally manipulated cognitive load (corroborated by pupillometry) and the mechanism for controlled behaviour by examining the relationship between frontal theta and motor cortical signals. We manipulated cognitive load requirements by having participants perform a combined Go/Switch pattern task, without explicit awareness of the presence of a stimulus pattern. The task effects of Go/Switch and pattern/deviant led to decreased RT, indicating pattern learning, while task related changes in PD corroborated parametric variations in cognitive load. Frontal theta was sensitive to these variations in cognitive load and was highly correlated with pupillometric activity, with weaker correlations between behaviour and motor cortical activity that did not survive correction for multiple comparisons. The correlations held across all trial types, including the unconsciously learned stimulus pattern, demonstrating that frontal control of behaviour may proceed without conscious awareness. Furthermore, cross-frequency coupling analysis revealed no coupling between frontal theta phase and sensorimotor gamma amplitudes (as hypothesised), demonstrating instead high amounts of ongoing phase synchrony between theta sources in bilateral middle frontal, right inferior frontal, and bilateral premotor areas. The implications for these findings are discussed below.

### Reaction Time Is Insufficient for Capturing Cognitive Control

The observation of equal beta ERD across trials within the left hemisphere would suggest that subjects prepared the Go response for every trial (Figure 6B). In contrast, reduced beta ERD within the right hemisphere for the Go trials compared with Sw trials indicates that subjects did not prepare the switch response during Go trials. Furthermore, similar beta ERD between PSw and DSw trials would suggest that subjects were able to quickly prepare and execute left-hand switch responses whether or not the switch was anticipated (Figure 6C). Taken together, given that Go responses were consistently prepared, it appears that for DGo trials any increased cognitive load would have been related to inhibiting the prepared Sw response, which lead to an observed increase in PD and theta responses, without a corresponding increase in RT.

Differences between RT and PD are congruent with our previous findings (Isabella et al., 2019). Increased PD has been associated with increased cognitive load (Kahneman and Beatty, 1966), however, without a corresponding increase in RT or decrease in performance efficiency, we interpret this finding of increased load without corresponding detectable behavioural effects as increased cognitive effort, occurring covertly. This finding has important implications for commonly used behavioural measures such as RT or efficiency in interpreting task difficulty or cognitive control, given that increasing cognitive effort need not produce detectable behavioural outcomes, as observed here. We propose that PD and frontal theta are more sensitive and more direct measures of cognitive control than RT, since PD and theta can capture differences in cognitive effort in circumstances requiring greater control.

### On the Relationship Between Brain Signals and Behaviour

Increased cognitive effort to maintain consistent RT across the two types of Go responses was likely driven by frontal theta activity. Although it did not survive correction for multiple comparisons in this study, a relationship between theta power and RT has been demonstrated previously (Gartner et al., 2015). This suggests that speeded responses may depend on theta signalling, and that it may be a finite resource. In addition, it is known that frontal theta increases are sensitive to the need for control processes (Jensen and Tesche, 2002; Cheyne et al., 2012). In the current study, frontal theta power was proportional to the amount of effort put into response inhibition and preparation, as demonstrated by a significant (*p* = 8.1 e^–9^) correlation with PD.

Given that most previous work on frontal theta focused on a single neural generator, the result of 5 distinct task-related frontal theta sources was of particular interest, given that the beamforming algorithm will tend to suppress temporally correlated activity in multiple brain regions. It is possible that this, combined with limitations of signal to noise in the theta frequency range resulted in one or more frontal sources to appear as multiple apparent sources. Although a thorough analysis to rule out this possibility is beyond the scope of this study, the current results are not consistent with such an interpretation. First, activity in each theta source demonstrated different sensitivity to the task parameters (Figure 5 and Table 1). Second, although mean dwPLI was consistently very high across all five sources and time points, wPLI as a measure is designed to be insensitive to source leakage (Vinck et al., 2011). Furthermore, if these connections were representing leakage from a single source it would be unlikely that their connection strength would modulate upwards and downwards with task parameters as they did in the current study (Figure 9). Lastly, recent work supports the existence of multiple (2–6) distinct and independent frontal theta sources during a Simon task (Töllner et al., 2017; Zuure et al., 2020). Therefore, we interpret these findings as representing multiple frontal sources of task related theta activity.

Differences in task effects across theta sources are worth noting. As was hypothesised, Right Middle Frontal cortex theta was sensitive to all task effects and correlated highly with cognitive load, as measured by PD (*r* = 0.96, Figure 8). Interestingly activity in the Right Premotor area was, in addition, similarly sensitive to cognitive load (*r* = 0.95). Given its proximity to the motor cortex, one would anticipate Right Premotor theta activity to be more closely linked with motor output. Indeed this was the case, as Right Premotor theta had a stronger correlation with RT and right hemisphere beta ERD than Right Middle Frontal theta. However, Right Premotor theta did not have a stronger correlation with contralateral gamma ERS than Right Middle Frontal theta did with gamma ERS. This finding is in line with our previous interpretation that sensorimotor gamma ERS may be involved in integrating cognitive signals into the motor cortex, rather than having a purely motor function (Isabella et al., 2015). Beyond these two theta sources, the other 3 sources (Left Middle Frontal, Right Inferior Frontal and Left Premotor) appear most sensitive to effects of deviations from the pattern (or, unexpected stimuli), and not sensitive to switching response hands. It is possible that these regions are more closely related to pattern learning in particular rather than cognitive processing in general.

We expected that frontal theta would be related to the need for inhibition, as indexed by ipsilateral beta ERD (Figure 6), and that theta would be inversely proportional to the extent of motor preparation, as indexed by contralateral beta ERD. Instead, it was found that frontal theta in the premotor cortex (D) was related to right hemispheric beta ERD across all trial types, which was in turn correlated with contralateral gamma ERS (Table 2). This would suggest that cognitive control of motor output may be a mix of unilateral and bilateral processes. This is in line with recent findings that tDCS over left sensorimotor cortex reduces left and right no-go errors, highlighting the interrelations between bilateral sensorimotor cortices (Friedrich and Beste, 2018). Furthermore, there is evidence for response conflict processing within the sensorimotor cortex (Coles et al., 1995; Cheyne et al., 2012), and it is hypothesised that gamma ERS may be important for resolving this conflict within the motor cortex contralateral to the executed movement. Thus, the current findings suggest that cognitive control mechanisms may not lie solely in the inhibition of a prepared response but could also involve the interplay between beta ERD preparatory processes and gamma ERS response monitoring processes across hemispheres in a bimanual task.

The interpretation that gamma ERS is important for resolving response conflict within the motor cortex is supported by previous work demonstrating that delayed sensorimotor gamma ERS predicted error responses (Isabella et al., 2015). When competing responses are not sufficiently resolved in order to update the motor plan prior to responding, an error may occur. This interpretation is supported by evidence in clinical populations. Kurz et al. (2014) demonstrated increased sensorimotor beta ERD and decreased gamma ERS in children with cerebral palsy who had difficulty anticipating grip forces, possibly related to deficits in motor planning (Kurz et al., 2014). We speculate given the current findings that impaired motor planning may be related to deficits in signalling from the frontal cortex, or inefficient integration into the sensorimotor cortex via beta and gamma activity. Although this study demonstrated a path between frontal theta and motor areas (i.e., SMA via theta phase coherence), it remains to be determined how these signals may be related to primary motor cortical beta and gamma signalling.

### On Frontal Theta Synchrony – An Unexpected Connection Between Theta and Motor Areas

Sensorimotor gamma ERS and frontal theta increased parametrically with cognitive control, and a correlation (that did not survive correction for multiple comparisons) was found between the two, suggesting a possible role for gamma in integrating theta activity into the motor cortex. Although PAC analysis did not reveal any significant coupling between any of the frontal theta sources and sensorimotor gamma ERS, given the relationship established between gamma and cognitive parameters, it is possible that an indirect relationship between frontal theta and motor gamma is mediated by beta ERD, or even via subcortical sources. Evidence to support this possibility is discussed below.

We were surprised to find 5 independent sources of frontal theta activity across both hemispheres that demonstrated phase coherence to various degrees (Figure 9). Previous work demonstrated the existence of multiple independent conflict-related theta sources with all-to-all granger causality, suggesting no “CEO-like” hierarchy but rather that these sources perform independent computations (Zuure et al., 2020). The current findings extend the previous results in demonstrating a mechanism for the functional link between theta sources was phase synchrony across specific sources of theta activity: bilateral middle frontal, right inferior frontal, and bilateral premotor areas. Importantly, the current findings link the more anterior frontal theta sources to premotor areas via theta functional connectivity, as well as correlations between activity in the premotor areas and primary motor cortices (Table 2).

It is notable that the current dwPLI results demonstrate very high baseline phase synchrony, with a relatively small task-related change. It is notable that previous studies that have used this measure found the same amount of task-related increases and decreases in dwPLI, although with much lower baseline levels (Babapoor-Farrokhran et al., 2017). Such high levels of ongoing theta phase synchrony might suggest an issue with dwPLI as a measure (e.g., too sensitive to spurious coupling), an issue with the task (e.g., causing too much spurious coupling), or it might suggest that these five frontal sources are part of an ongoing “task-on” theta network. In support of this latter possibility, the middle frontal gyrus has been implicated in the frontoparietal (executive control) network that is associated with performance of cognitive tasks (Seeley et al., 2007). Thus, the present results suggest that this network may be supported by ongoing frontal theta phase synchrony throughout task performance.

### Summary and Conclusion

Frontal theta has been lauded as the ‘lingua franca’ for cognitive control (Cavanagh and Frank, 2014). This assertion has, however, lacked sufficient evidence of the required link between cognitive control and behaviour. Based on previous literature we hypothesised that frontal theta would mediate cognitive control via theta-gamma cross-frequency coupling from the frontal to primary motor cortices, the strength of which should vary with cognitive effort.

The current study provides evidence for a link between frontal theta and current best measures of cognitive control (i.e., PD) across parametric variations, as well as a basis for interactions among theta sources in frontal and premotor areas that lead to behavioural output, via a different mechanism than hypothesised. We found evidence to support frontal theta as a mechanism for executive control of behaviour via interregional theta phase synchrony, thus linking activity between frontal and motor areas via theta oscillatory phase synchrony.

It has been suggested that the same neural substrates support conscious and unconscious processes via the same neural computations, where the difference between the two might only be a matter of degree (Horga and Maia, 2012). Others have gone further to suggest that there is no causal role for conscious processes in action control, and that automatic processes may underlie normal motor behaviour that is generally attributed to top-down cognitive control (Kunde et al., 2012; McBride et al., 2012; Hommel, 2013; Jasinska, 2013). Current understanding of implicit learning suggests that it is also automatically acquired and not under cognitive control. That subjects in the current study were not consciously aware of the existence of a pattern suggests that they were automatically increasing effort required to inhibit responses, either when predicted as in PSw or when unpredicted as in DSw. The association of frontal theta power and phase with inhibitory control, in the current study as well as in others, suggests that both inhibitory control and processing of other parameters associated with cognitive effort such as response selection may all be under unconscious control.

## Data Availability Statement

The raw data supporting the conclusions of this article will be made available by the authors upon reasonable request, without undue reservation.

## Ethics Statement

The studies involving human participants were reviewed and approved by Hospital for Sick Children Research Ethics Board. The patients/participants provided their written informed consent to participate in this study.

## Author Contributions

SI conceived of and designed the study, collected and analysed the data, and wrote the manuscript. DC contributed funding to support the study. All authors contributed to manuscript revision, read, and approved the submitted version.

## Conflict of Interest

The authors declare that the research was conducted in the absence of any commercial or financial relationships that could be construed as a potential conflict of interest.

## Publisher’s Note

All claims expressed in this article are solely those of the authors and do not necessarily represent those of their affiliated organizations, or those of the publisher, the editors and the reviewers. Any product that may be evaluated in this article, or claim that may be made by its manufacturer, is not guaranteed or endorsed by the publisher.

## Footnotes

1
www.neurobs.com

2
https://www.fieldtriptoolbox.org/
