# Supplementary material for: Inhibitory Control in the Absence of Awareness: Interactions Between Frontal and Motor Cortex Oscillations Mediate Implicitly Learned Responses
Source: Front Hum Neurosci. 2021 Dec 22;15:786035. doi: 10.3389/fnhum.2021.786035 (PMC8727746; doi:10.3389/fnhum.2021.786035)
Supplement: Supplementary file 1 [file Table_1.docx]

**Supplementary Table 1. ANOVA results of task-related changes in dwPLI across source pairs.**

|  | | **Main Effects** | | **Interactions** |
| --- | --- | --- | --- | --- |
| **Connected Regions** | **Mean**  **task-evoked dwPLI** | **Switch** | **Pattern** | **Switch*Pattern** |
| A: R Middle Frontal –  B: L Middle Frontal | - 0.008 | n/s | F(1,15) = 10.99,  p = 0.005,  η_p_^2^  = 0.422 | n/s |
| A: R Middle Frontal –  C: R Inferior Frontal | - 0.008 | F(1,15) = 6.924,  p* = 0.0189 | F(1,15) = 30.98,  p < 0.001,  η_p_^2^  = 0.674 | F(1,15) = 10.65,  p = 0.005,  η_p_^2^  = 0.415 |
| A: R Middle Frontal –  D: R Precentral | - 0.011 | F(1,15) = 32.93,  p < 0.001,  η_p_^2^  = 0.687 | F(1,15) = 27.23,  p < 0.001,  η_p_^2^  = 0.645 | F(1,15) = 36.17,  p < 0.001,  η_p_^2^  = 0.707 |
| A: R Middle Frontal –  E: L Precentral | 0.013 | 0.014 | F(1,15) = 78.12,  p < 0.001,  η_p_^2^  = 0.839 | n/s |
| B: L Middle Frontal –  C: R Inferior Frontal | 0.005 | 0.0271 | n/s | n/s |
| B: L Middle Frontal –  D: R Precentral | 0 | n/s | n/s | n/s |
| B: L Middle Frontal –  E: L Precentral | 0.003 | n/s | n/s | n/s |
| C: R Inferior Frontal –  D: R Precentral | 0.011 | n/s | F(1,15) = 18374,  p < 0.001,  η_p_^2^  = 0.555 | n/s |
| C: R Inferior Frontal –  E: L Precentral | - 0.01 | F(1,15) = 18.37,  p < 0.001,  η_p_^2^  = 0.550 | F(1,15) = 40.12,  p < 0.001,  η_p_^2^  = 0.728 | F(1,15) = 12.99,  p = 0.003,  η_p_^2^  = 0.464 |
| D: R Precentral –  E: L Precentral | - 0.004 | n/s | F(1,15) = 15.08,  p = 0.001,  η_p_^2^  = 0.501 | n/s |

Negative dwPLI values denote a *decrease* in task-related oahse coherence. * not significant after Holm-adjustment.
